# Supplementary material for: lnc-SAMD14-4 can regulate expression of the COL1A1 and COL1A2 in human chondrocytes
Source: PeerJ. 2019 Sep 2;7:e7491. doi: 10.7717/peerj.7491 (PMC6727836; doi:10.7717/peerj.7491)
Supplement: Supplemental Information 1 [file peerj-07-7491-s001.docx]

**Miame Checklist**

Part 1 Experiment description

-         **experiment type: human**

-         **experimental variables (osteoarthritis vs. non-osteoarthritis)**

-         **n-count: 5 vs 5**

-         **tissues used for slide : articular cartilage chondrocytes**

-         **mouse age, and other variables :** the mean age of the 5 OA patients used for microarray is 63 years; the mean age of the 5 control patients used for microarray is 28.8 years;

Part 2 Array design.

*“**The aim of this section is to provide a systematic definition of all arrays used in the experiment, including the genes represented and their physical layout on the array.”*

*“The array-type definition includes information common to all arrays of a particular type (such as glass-slide spotted with PCR-amplified cDNA clones) as well as precise descriptions of the physical content of each element (spot or feature). This section consists of three parts: (i) a description of the array as a whole (such as platform type, provider and surface type); (ii) a description of each type of element or spot used (properties that are typically common to many elements, such as 'synthesized oligo-nucleotides' or 'PCR products from cDNA clones'); and (iii) a description of the specific properties of each element, such as the DNA sequence and, possibly, quality-control indicators.”*

-         **Array series:** OE Biotech Human Microarray lncRNA (Affymetrix)

-         **Deconvoluted spot list with gene names:** The microarray data are available through the GEO database with accession number GSE113825.

-         **Array type (mouse, human, cDNA, oligo, number of genes):**

Human lncRNA microarray, the array included 63,542 non-coding and 27,134 coding transcripts

-         **Array size:**

-         **Slide type (and coating):**

Part 3 Samples

*“The MIAME 'sample' concept represents the biological material (or biomaterial) for which the gene expression profile is being established. This section is divided into three parts which describe the source of the original sample (such as organism taxonomy and cell type) and any biological*in vivo*or*in vitro*treatments applied, the technical extraction of the nucleic acids, and their subsequent labeling.”*

-         **Cy3/Cy5 labels for tissues:** Cyanine-3 (Cy3)

-         **Dye swap? Or reference control?**

-         **Labelling protocol used** :Cyanine-3 (Cy3) labeled cRNA was prepared from 0.2 μg RNA using the One-Color Low RNA Input Linear Amplification PLUS kit (Agilent) according to the manufacturer's instructions, followed by RNAeasy column purification (QIAGEN, Valencia, CA). Dye incorporation and cRNA yield were checked with the NanoDrop ND-1000 Spectrophotometer.

-         **Sample extraction protocol used:** RNA was extracted by mirVanaTM RNA Isolation Kit (Applied Biosystem p/n AM1556 ) following the manufacturer's instructions.

-         **Amount of sample labelled: 10 tissues**

Part 4 Hybridizations

*“This section defines the laboratory conditions under which the hybridizations were carried out. Other than a free-text description of the hybridization protocol, MIAME requires that a number of critical hybridization parameters are explicitly specified: choice of hybridization solution (such as salt and detergent concentrations), nature of the blocking agent, wash procedure, quantity of labeled target used, hybridization time, volume, temperature and descriptions of the hybridization instruments.”*

-         **Hybridization protocol:** 0.6 μg of Cy3-labelled cRNA (specific activity >10.0 pmol Cy3/μg cRNA) was fragmented at 60°C for 30 minutes in a reaction volume of 22.5μl containing 1x Agilent fragmentation buffer and 2x Agilent blocking agent following the manufacturers' instructions. On completion of the fragmentation reaction, 22.5μl of 2x Agilent hybridization buffer was added to the fragmentation mixture and hybridized to OE Biotech Human Microarray lncRNA (Affymetrix)（1*K）for 17 hours at 65°C in a rotating Agilent hybridization oven. After hybridization, microarrays were washed 1 minute at room temperature with GE Wash Buffer 1 (Agilent) and 1 minute with 37°C GE Wash buffer 2 (Agilent), then dried immediately by brief centrifugation.

-         **ALL modifications and deviations from the protocol**

-         **Manual hybridization or automatic chamber?:**

-         **Number of slides done at the same time**

-         **Hyb time**

-         **Number of washes**

-         **Amount of labelled sample hybridized: 10**

-         **Labelling efficiency**

Part 5 Measurements

*“Image data should be provided as raw scanner image files (such as TIFF), accompanied by scanning information that includes relevant scan parameters and laboratory protocols.”*

-         **Which version of scanner software used:** Feature Extraction Software 10.7.1.1 (Agilent)

-         **Laser power for scan**

-         **Instrument model numbers**

-         **Must save original .tiff format images (****composite image is optional)**

*For each experimental image, a microarray quantification matrix contains the complete image analysis output as directly generated by the image analysis software (normally provided as separate spreadsheet-type files). Note that for a given image this is a 2D matrix, where array elements (spots or features) constitute one dimension and quantification types (such as mean and median intensity, mean or median background intensity) are the second dimension.*

-         **Normalization protocol**

-         **Does the scanner software subtract background? How much?**

-         **Spot raw values, background intensity, ch1 and 2 intensity, etc.**

-         **Corresponding gene name**

-         **Methods of analysis (MAN, Spotfire, Genespring) be detailed.**

-         **Normalized to controls? Controls removed? All normalization parameters**

-         **Name of Images, Experiment, and location of files.**

-         **Lowess or other normalization if used (and parameters)**

*Finally, the gene expression matrix (summarized information) consists of sets of gene expression levels for each sample. If microarray quantification matrices can be considered spot/image centric, then the gene expression matrix is gene/sample centric. At this point, the expression values may have been normalized, consolidated and transformed in any number of ways by the submitter in order to present the data in a form amenable to scientific analysis. Rather than attempting to impose a standard for gene expression values, MIAME indicates preferred detailed specifications of all numerical calculations applied to unprocessed quantifications in (b) that have led to the data in (c). Experimenters are encouraged, though not required, to provide reliability indicators (such as s.d.) for each data point.*

-         **Output file**

-         **Normalized ratios**

-         **Numerical manipulations**

-         **Cut off values**

Part 6 Normalization controls

*“A typical microarray experiment involves a number of hybridization assays in which the data from multiple samples are analyzed to identify relative changes in expression levels, identify differentially expressed genes and, in many cases, discover classes of genes or samples having similar patterns of expression.”*

-         **Hypothesis**

-         **Gene expression patterns found**

-         **Controls used, normalization methods used (see above)**
