# Supplementary material for: lnc-SAMD14-4 can regulate expression of the COL1A1 and COL1A2 in human chondrocytes
Source: PeerJ. 2019 Sep 2;7:e7491. doi: 10.7717/peerj.7491 (PMC6727836; doi:10.7717/peerj.7491)
Supplement: Figure S1 — (A) the SDS-PAGE gels of total RNA. 28S ribosomal RNA and 18S ribosomal RNA bands appeared in all tissue samples (B) The electrophoresis pattern of SDS-PAGE of total RNA. (C) the analysis of SDS-PAGE of total RNA, RNA Integrity Number(RIN) >7 represent the quality of RNA can satisfy the study requirements. [file peerj-07-7491-s002.pdf]

A

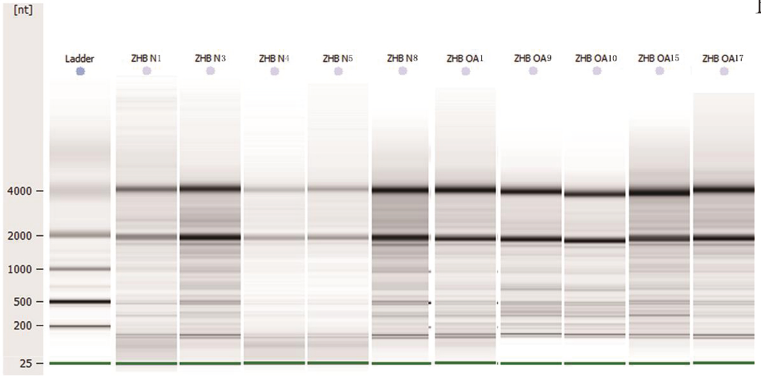

B

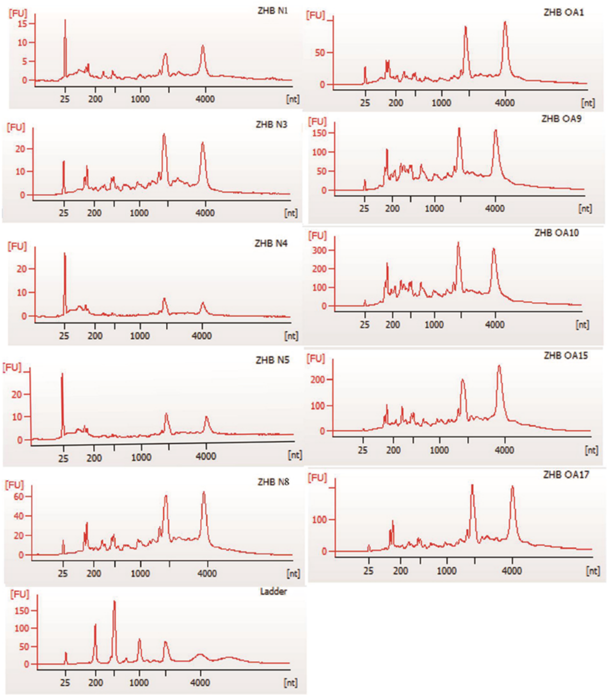

C

No	Sample No	concentration (pg/μl)	Volume (μl)	Total quantity (pg)	2100 28S/18S	2100 RIN	result
1	N1	110	20	2200	1.3	7.1	pass
2	N3	270	20	5400	1.2	7.0	pass
3	N4	105	45	4725	0.9	7.1	pass
4	N5	110	45	4950	0.9	7.7	pass
5	N8	741	20	14820	1.3	7.0	pass
6	OA1	877	45	39465	1.5	7.5	pass
7	OA9	818	20	16360	1.5	7.1	pass
8	OA10	1534	20	30680	1.3	7.3	pass
9	OA15	2678	20	53560	1.7	7.1	pass
10	OA17	1917	45	86265	1.2	7.2	pass
