# Supplementary material for: lnc-SAMD14-4 can regulate expression of the COL1A1 and COL1A2 in human chondrocytes
Source: PeerJ. 2019 Sep 2;7:e7491. doi: 10.7717/peerj.7491 (PMC6727836; doi:10.7717/peerj.7491)

UCSC Genome Browser on Human Feb. 2009 (GRCh37/hg19) Assembly

move <<< << < > >> >>> zoom in 1.5x 3x 10x base zoom out 1.5x 3x 10x 100x

chr7:50,187,607-50,188,784 1,178 bp. enter position, gene symbol, HGVS or search terms go

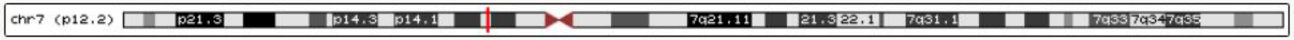

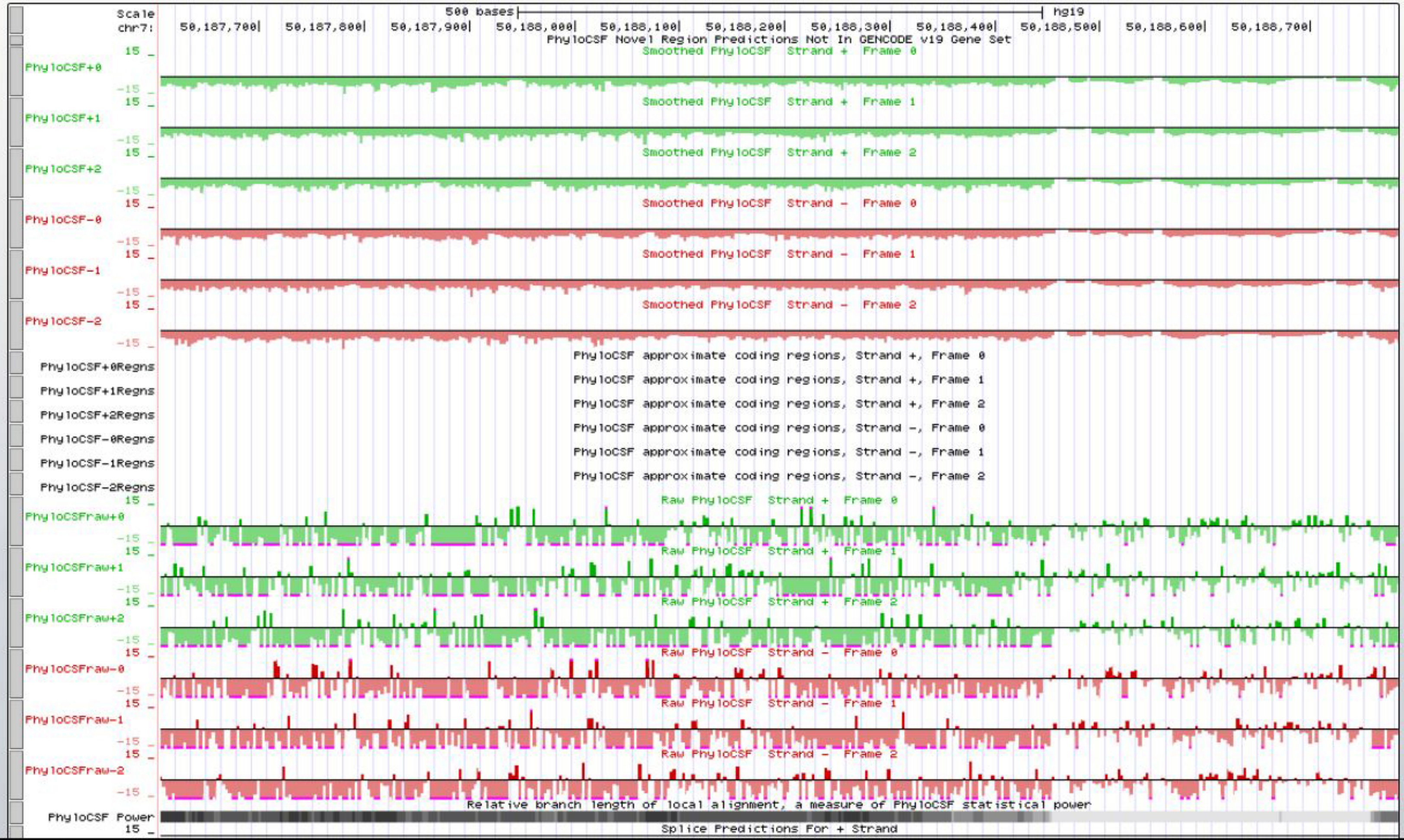

Supplement: Figure S3 [file peerj-07-7491-s004.pdf]
