# Supplementary figures and images for: lnc-SAMD14-4 can regulate expression of the COL1A1 and COL1A2 in human chondrocytes
Source: PeerJ. 2019 Sep 2;7:e7491. doi: 10.7717/peerj.7491 (PMC6727836; doi:10.7717/peerj.7491)

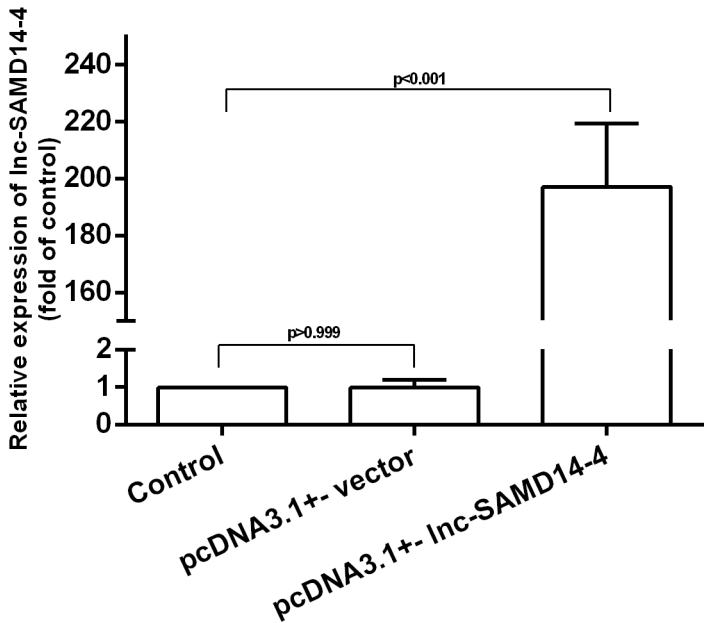

Supplement: Figure S4 — QPCR analysis confirmed increased lnc-SAMD14-4 expression in human primary chondrocytes after pcDNA 3.1+-lnc-SAMD14-4 transfection. [file peerj-07-7491-s005.pdf]

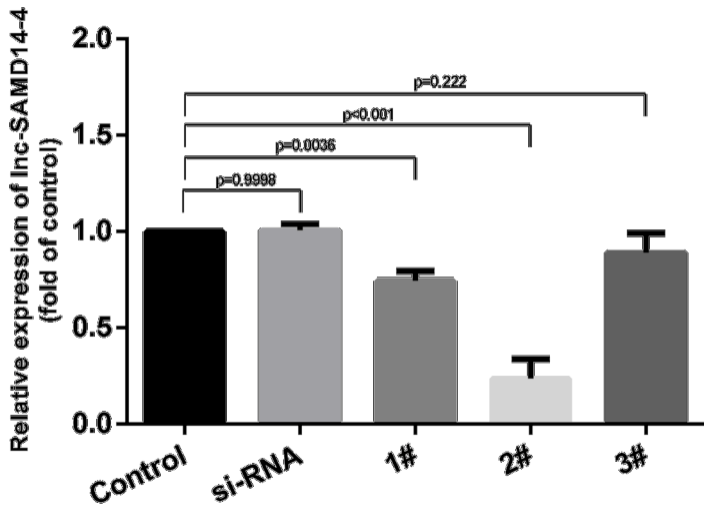

Supplement: Figure S5 — The strongest inhibitory result was achieved by lnc-SAMD14-4(2#) [file peerj-07-7491-s006.pdf]
