# Supplementary material for: lnc-SAMD14-4 can regulate expression of the COL1A1 and COL1A2 in human chondrocytes
Source: PeerJ. 2019 Sep 2;7:e7491. doi: 10.7717/peerj.7491 (PMC6727836; doi:10.7717/peerj.7491)
Supplement: Table S2 [file peerj-07-7491-s008.doc]

Gene name forward primers(5’-3’) reverse primers(5’-3’)

lnc-SAMD14-4 AGAGTGGTGATCGTGGTGAG TAAGGCCCTGACATCTTGCA

lnc-MARCKS-7 TGTCTACAGTGCGGCTACAA GTGGTGGAGAGGACAGAACA

lnc-MSMP-2 TGCTAGACAGGCCTTCGAA GGAGCCAAGACTCACCCAG

lnc-NPVF-4 CCTCCTAGATGACCTGCCTG TCAGTCTTTCAGAGGGGCAG

SERPINF1 TCCAGGGAGCAGAAAAGAGG GCTTGTTCACGGGGACTTTG

COL1A1 CCCTCCCACAACTCTGACAT AGGGAATCGCGTTTATAGGGT

COL1A2 GAGGGCAACAGCAGGTTCACTTA TCAGCACACCGATGTCCAA

MYOC AGGTTCTTCTGTGCACGTTG GGTGCTGCTGTCTCTCTGTA PAK3 AGATGCTCTCACACACCCAG ATAGGAGATCACAGCCGCAG

GAPDH CAAGGTCATCCATGACAACTTTG GTCCACCACCCTGTTGCTGTAG
