# Supplementary material for: lnc-SAMD14-4 can regulate expression of the COL1A1 and COL1A2 in human chondrocytes
Source: PeerJ. 2019 Sep 2;7:e7491. doi: 10.7717/peerj.7491 (PMC6727836; doi:10.7717/peerj.7491)
Supplement: Figure S2 [file peerj-07-7491-s003.pdf]

A

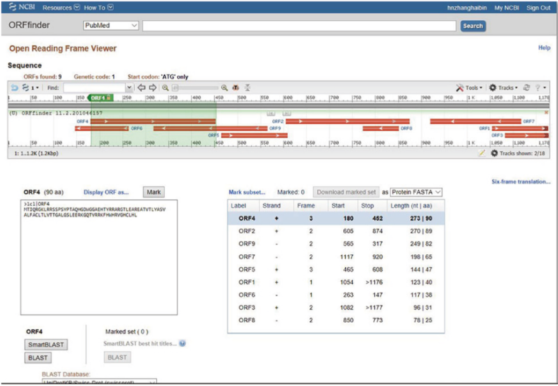

NCBI
Resources
How To
hnzhanghaibin
My NCBI
Sign Out
ORFfinder
PubMed
Search
Open Reading Frame Viewer
Help
Sequence
ORFs found: 9
Genetic code: 1
Start codon: 'ATG' only
Find:
Tools
Tracks
ORF4
ORFfinder 11.2.201046157
ORF2
ORF6
ORF9
ORF8
ORF7
ORF1
ORF5
ORF3
1: 1..1.2K (1.2Kbp)
Tracks shown: 2/18
Six-frame translation...
ORF4 (90 aa)
Display ORF as...
Mark
>lcl|ORF4
MTIQRGKLRRSSPSYPTAQHGDWGGAEHTYRRARGTLEAREATVTLYASV
ALFACLTLVTTGALGSLEERKGQTVRRKFHWHRVGHCLHL
Mark subset...
Marked: 0
Download marked set
as Protein FASTA
Label | Strand | Frame | Start | Stop | Length (nt | aa)
ORF4 | + | 3 | 180 | 452 | 273 | 90
ORF2 | + | 2 | 605 | 874 | 270 | 89
ORF9 | - | 2 | 565 | 317 | 249 | 82
ORF7 | - | 2 | 1117 | 920 | 198 | 65
ORF5 | + | 3 | 465 | 608 | 144 | 47
ORF1 | + | 1 | 1054 | >1176 | 123 | 40
ORF6 | - | 1 | 263 | 147 | 117 | 38
ORF3 | + | 2 | 1082 | >1177 | 96 | 31
ORF8 | - | 2 | 850 | 773 | 78 | 25
ORF4
SmartBLAST
BLAST
Marked set (0)
SmartBLAST best hit titles...
BLAST
BLAST Database:

B

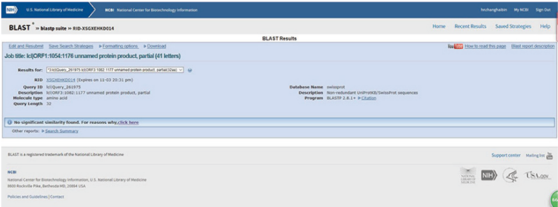

U.S. National Library of Medicine
NCBI National Center for Biotechnology Information
hnzhanghaibin
My NCBI
Sign Out
BLAST » blastp suite » RID-XSGXEHKD014
Home
Recent Results
Saved Strategies
Help
BLAST Results
Edit and Resubmit
Save Search Strategies
Formatting options
Download
How to read this page
Blast report description
Job title: lcl|ORF1:1054:1176 unnamed protein product, partial (41 letters)
Results for:
RID XSGXEHKD014 (Expires on 11-03 20:31 pm)
Query ID lcl|Query_261975
Description lcl|ORF3:1082:1177 unnamed protein product, partial
Molecule type amino acid
Query Length 32
Database Name swissprot
Description Non-redundant UniProtKB/SwissProt sequences
Program BLASTP 2.8.1+ Citation
No significant similarity found. For reasons why, click here
Other reports: Search Summary
BLAST is a registered trademark of the National Library of Medicine
Support center
Mailing list
NCBI
National Center for Biotechnology Information, U.S. National Library of Medicine
8600 Rockville Pike, Bethesda MD, 20894 USA
Policies and Guidelines | Contact
